# Proposal for the development of an international minimal data collection for juvenile dermatomyositis (JDM)

**DOI:** 10.1186/1546-0096-9-S1-P51

**Published:** 2011-09-14

**Authors:** Liza J McCann, Clarissa Pilkington, Laura Beard, Angelo Ravelli, Adam Huber, Lucy R Wedderburn

## Background

There are a variety of groups collecting prospective data on patients with juvenile idiopathic inflammatory myopathies (IIM), including the UK JDM Cohort Biomarker Study and Repository^1^, CARRA, and Euromyositis. Datasets are partially overlapping. The development of a consensus minimal data collection would facilitate comparison and communication between these groups.

## Aim

Proposal of a new international minimal data collection, potentially valuable in trials and clinical contexts, to be collected by clinicians for all JDM patients, respecting data protection and ownership.

## Methods

A comparison was made of current variables collected within the UK JDM Cohort / Biomarker Study, CARRA, Euromyositis and those used in a multi-national inception study of 27 centres in Europe / Latin America.^2^ Variables common to at least 2 datasets were considered for inclusion, based on agreement between collaborators.

## Results

The authors propose a minimum data collection for JDM patients, achievable by clinicians within their current practice. A more detailed collection of activity / damage indices could be performed in specialist / research environments.

## Conclusion

Development of an international minimal data collection for use in trials would allow greater understanding of disease course and prognosis, enhance international collaboration between groups, and facilitate linking to biobanks. The proposed dataset would require testing through existing collaborations (IMACS, PRINTO and others). Collaboration with adult groups (eg. via Euromyositis) may allow harmonised data collection from paediatric to adult services, providing valuable outcome data for this rare disease.

## References

[B1] Martin N Krol P Smith S A national registry for juvenile dermatomyositis and other paediatric idiopathic inflammatory myopathies: 10 years' experience; the Juvenile Dermatomyositis National (UK and Ireland) Cohort Biomarker Study and Repository for Idiopathic Inflammatory Myopathies Rheumatology (Oxford) 2011 50 1 137 45 10.1093/rheumatology/keq261 PMC2999955 20823094

[B2] Ravelli A Trail L Ferrari C Long-term outcome and prognostic factors of juvenile dermatomyositis: a multinational, multicenter study of 490 patients Arthritis Care Res (Hoboken) 2010 62 1 63 72 10.1002/acr.20015 20191492

